# The dragonflies in the collection of Sapienza University of Rome

**DOI:** 10.3897/BDJ.13.e163929

**Published:** 2025-09-30

**Authors:** Davide Tamagnini, Pierfilippo Cerretti, Naomi De Leo, Claudio Chimenti

**Affiliations:** 1 Department of Biology and Biotecnology Charles Darwin, Sapienza University of Rome, Rome, Italy Department of Biology and Biotecnology Charles Darwin, Sapienza University of Rome Rome Italy

**Keywords:** biodiversity data, entomology, geospatial data, insect collection, Italy, natural history museums, specimen digitisation

## Abstract

**Background:**

The Odonata (dragonflies and damselflies) are key bioindicators of freshwater ecosystem health and are widely used in ecological and conservation research. Historical entomological collections provide a valuable source of biodiversity data, particularly for tracking species distributions over time and detecting environmental changes. The Odonata collection of the Museum of Zoology at Sapienza University of Rome includes 2406 specimens, representing 77 taxa (74 species and three identified at genus level) across 33 genera and 11 families. Most specimens were collected in Italy, with a small subset from other parts of Europe. This collection forms part of the broader entomological heritage of the University and is now being preserved and made accessible for research and public engagement through digitisation and online dissemination initiatives.

**New information:**

This is the first publicly available, digitised dataset of the Odonata collection from the Museum of Zoology at Sapienza University of Rome (MZUR), comprising 2406 specimens. All records have been georeferenced and standardised following the Darwin Core format. The dataset is published on the Global Biodiversity Information Facility (GBIF) under the MZUR institutional profile, significantly increasing its visibility and accessibility. In addition to data publication, the digitisation process included high-resolution photography of specimens and the transcription of original label data. An interactive web-based platform was also developed using Python-based tools, enabling dynamic exploration of the collection and enhancing its use for research, education and outreach.

## Introduction

Numerous studies have highlighted the importance of digitising entomological collections to support the conservation and analysis of biodiversity (Beaman and Cellinese 2012; Deflorian et al. 2020). While many museums and herbaria maintain catalogues of their collections, these are often still in paper format, making them susceptible to deterioration and difficult to access. To address these limitations, many institutions are transitioning to digital Collection Management Systems (CMS), which not only preserve data more securely, but also enable the integration of detailed metadata, including specimen images, taxonomic information, collector names, collection dates and geographic locations (Carpinone 2010).

Digital cataloguing offers a structured and systematic overview of collections, allowing for easy aggregation of records by species, region or collection period, even when specimens are housed in different physical locations (Baird 2010). Furthermore, digitisation facilitates comparisons across collections and enhances opportunities for collaborative research and comparative analysis. A significant advantage is the ability to georeference records automatically, supporting integration with biodiversity databases and the development of species distribution models (Nelson and Ellis 2018a).

Digitisation is also essential for the documentation and long-term preservation of physical specimens, which are vulnerable to degradation caused by mould, humidity and pests such as psocids (Psocoptera) and carpet beetles (*Anthrenus* spp.), posing a constant threat to their integrity (Pinniger 2010). Effective preventative conservation requires strict environmental control - particularly humidity regulation - to inhibit the proliferation of biodeterioration agents (Florian 1997). For Odonata, proper post-capture treatment is crucial to preserve both morphological integrity and the distinctive colouration of each species, which can fade over time without adequate conservation measures (Tennessen 2019).

In this context, digitisation not only safeguards the information contained in entomological collections, but also enhances access to data for a wider research community. The creation of digital archives enables long-term monitoring and comparative studies without the need for repeated physical handling, thereby minimising the risk of specimen damage (Beaman and Cellinese 2012; Blagoderov et al. 2012; Nelson and Ellis 2018a). This approach promotes accessibility and fosters new research in taxonomy, ecology and biogeography.

Against this backdrop, we have digitised the Odonata collection, housed at the Museum of Zoology (MZUR) of Sapienza University of Rome, comprising 2406 specimens. Odonata (dragonflies and damselflies) is an order of aquatic insects characterised by an amphibious life cycle and incomplete metamorphosis (hemimetabolous development). The larvae live in both lentic and lotic freshwater environments, where they prey on small invertebrates and occasionally fish fry. Adults are skilled fliers and efficient predators of flying insects. All life stages play important ecological roles and are widely recognised as bioindicators of environmental quality, since their presence and diversity reflect the health of freshwater habitats (Miguel et al. 2017).

In Italy, approximately 95 Odonata species are currently known, belonging to two suborders: Zygoptera (damselflies) and Anisoptera (true dragonflies). Anisoptera rest with wings spread horizontally, while Zygoptera - characterised by slender bodies, large eyes and short bristle-like antennae - fold their wings over the abdomen when at rest. Italian Odonata inhabit a wide range of environments, from coastal wetlands to alpine streams and several species are of particular conservation concern. Monitoring their populations is becoming increasingly relevant to assess the impacts of climate change and anthropogenic pressure on aquatic ecosystems (Miguel et al. 2017).

The main objective of this work is to describe the digitisation and online publication of this historical entomological collection and to highlight its importance for future research on biodiversity and the distribution of Odonata. This initiative is part of a broader effort to enhance historical natural history collections, which are now widely acknowledged as essential resources for studying biodiversity patterns and environmental changes over time (Suarez and Tsutsui 2004), for establishing new digitisation standards in museums (De Leo et al. 2025) and for supporting conservation efforts (Meineke et al. 2018). Our study contributes to the knowledge of European dragonflies, promotes the use of museum data in ecological and biogeographical research and encourages further digitisation and open sharing of natural history collections.

Although most specimens were collected in Italy, the dataset also includes samples from other European regions. All specimens have been photographed and the information from their labels has been digitised and made available via the Global Biodiversity Information Facility (GBIF) (Robertson et al. 2014; Nelson and Ellis 2018b), ensuring full accessibility to the scientific community.

## General description

### Purpose

This dataset presents the digitised collection of Odonata (dragonflies and damselflies), curated at the Zoological Museum of the University of Rome "La Sapienza". It includes 2406 specimens collected between 1936 and 2009, primarily from Italy, but also from other parts of Europe. The collection covers 73 full species names and three generic names belonging to 11 families and 33 genera, representing over 90% of the currently known Italian odonatofauna (Carchini et al. 1985, La Porta et al. 2023). Each specimen is accompanied by detailed label data, including collection date, locality, collector and identifier and is fully georeferenced. The digital archive, built using a series of custom Python scripts, provides high-resolution images and searchable metadata for each specimen. This dataset is a valuable resource for studies on biodiversity, biogeography, conservation and long-term ecological trends.

### Additional information

- The digitisation process involved high-resolution photography and label transcription for each specimen;
- All images were standardised through automated cropping and calibration procedures using Photoshop and Python scripts;
- The specimens are physically stored at the Zoological Museum of Sapienza University of Rome under controlled environmental conditions to prevent deterioration;
- A custom-built HTML interface allows users to browse the collection interactively, filter by species, locality, identifier and other metadata fields;
- The digital archive is available online;
- This dataset contributes to the goals of the National Biodiversity Future Centre (NBFC), supporting biodiversity research and conservation in Italy.

## Project description

### Title

The dragonflies in the collection of Sapienza University of Rome

### Personnel

- **Project coordinator**: Claudio Chimenti;
- **Data digitisation**: Davide Tamagnini, Pierfilippo Cerretti, Naomi De Leo, Claudio Chimenti;
- **Software and script development**: Claudio Chimenti;
- **Photography and image processing**: Claudio Chimenti.

### Study area description

The collection includes Odonata specimens mainly from Italy, particularly from the Lazio, Apulia and Tuscany regions, as well as a smaller number from other European countries. The dataset reflects decades of fieldwork conducted between 1936 and 2009, encompassing a broad variety of freshwater habitats.

### Design description

The primary goal of this project was the complete digitisation and web publication of the historical Odonata collection, housed at the Zoological Museum of Sapienza University of Rome. This effort supports biodiversity research, fosters open data sharing and contributes to long-term environmental monitoring by making valuable entomological data openly accessible.

The collection includes whole specimens mounted on cards that contain essential metadata, such as the collector’s name (who often also served as the identifier), collection locality, date and specimen sex. Additional annotations sometimes accompany the labels, highlighting particular traits or ecological context. To ensure long-term preservation, each specimen and its label were placed in a protective entomological envelope, shielding it from external agents and minimising the risk of deterioration (Pinniger 2010). This standardised procedure not only preserves the physical integrity of the specimens, but also facilitates digitisation, allowing integration into scientific databases (Beaman and Cellinese 2012). In cases where a male and female were collected together, both were stored in the same envelope, resulting in a slightly higher number of specimens than the 2,406 recorded entries.

Each envelope was assigned a unique identifier composed of three parts: a fixed prefix denoting the institution (MZUR), a code for the Odonata collection (ODOB) and a sequential five-digit number. This standardised coding system guarantees unambiguous specimen referencing across both physical and digital records.

As already described by Piervittori et al. (2025), the necessary information for uploading the collection data to the GBIF platform is presented in the subsequent section named Data resources.

**Figure 1.**
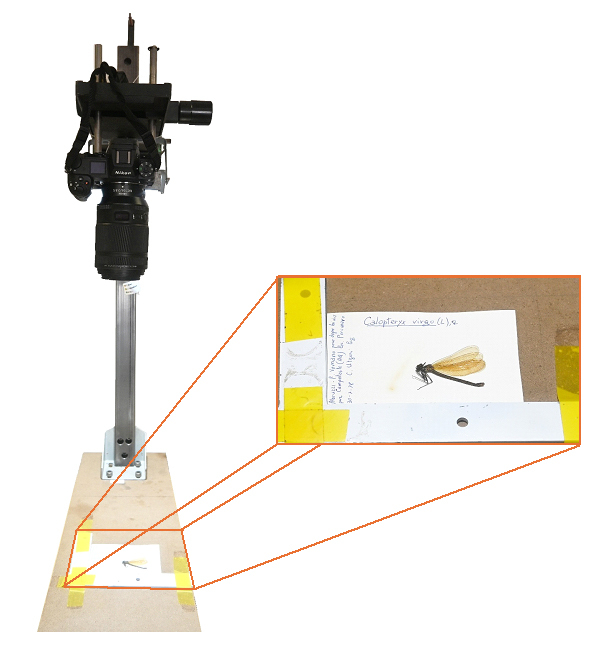
The camera mounted on the tripod and a detail of the frame used to position the specimen on its label.

Specimens were digitised through high-resolution photography using a Nikon Z7 camera mounted on a tripod and equipped with a fixed-focus Nikkor MC 105/2.8S lens. A frame positioned at the base of the set-up ensured consistent placement of labels during shooting (Fig. 1). Due to the mismatch between label size (12.4 × 7.4 cm) and camera frame ratio (8256 × 5504 pixels), images were cropped and resampled to focus on the label and specimen area. Although this process occasionally excluded parts of handwritten annotations from the images, all information was manually transcribed and preserved in digital files, ensuring complete data integrity.

To organise and integrate the digitised material with the corresponding label metadata, a Python script was developed to extract image filenames from a designated folder and list them in an Excel sheet. This allowed for reliable association between photographic files and metadata recorded separately.

In addition, a suite of five Python scripts was developed to build an interactive and navigable digital archive of the collection, accessible at this link.

**Figure 2.**
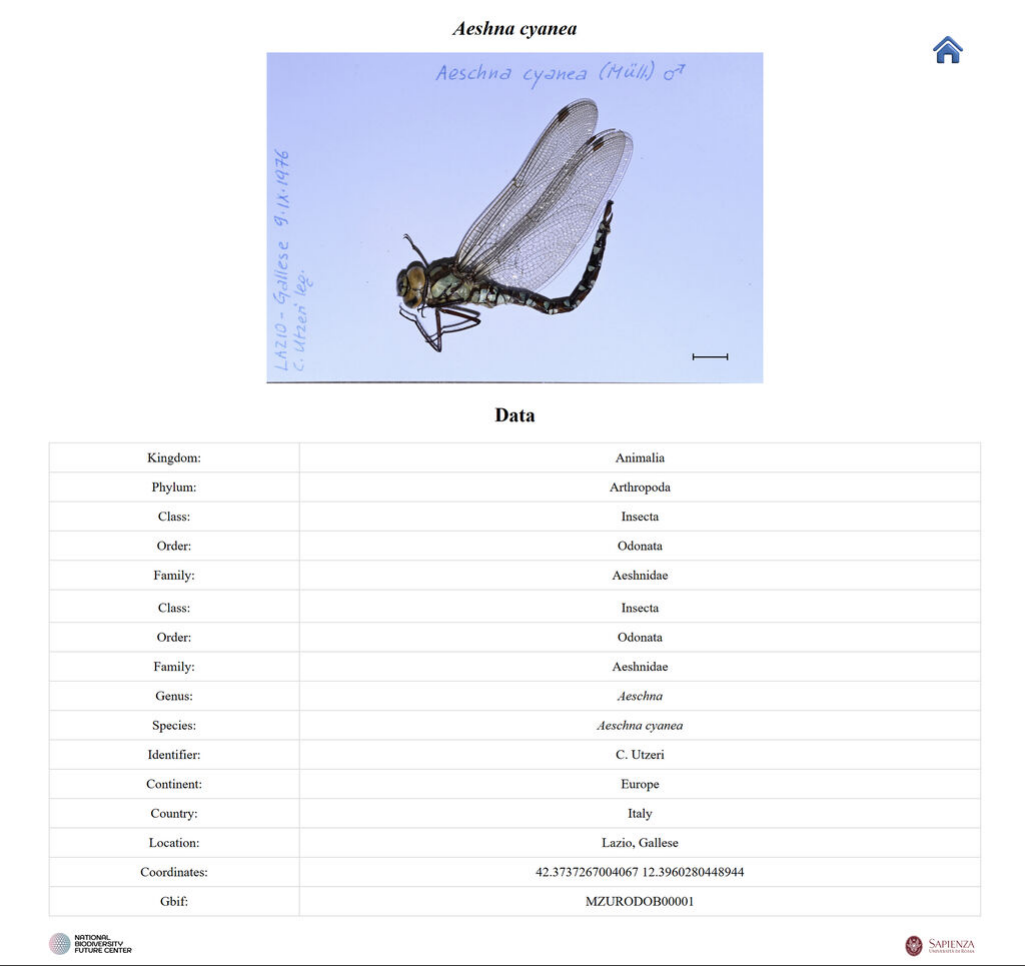
The web page for the single specimen.

**First script**: Generates an individual HTML page for each specimen, named according to the species and populated with key label data retrieved automatically from the corresponding row in the Excel file (Fig. 2).

**Figure 3.**
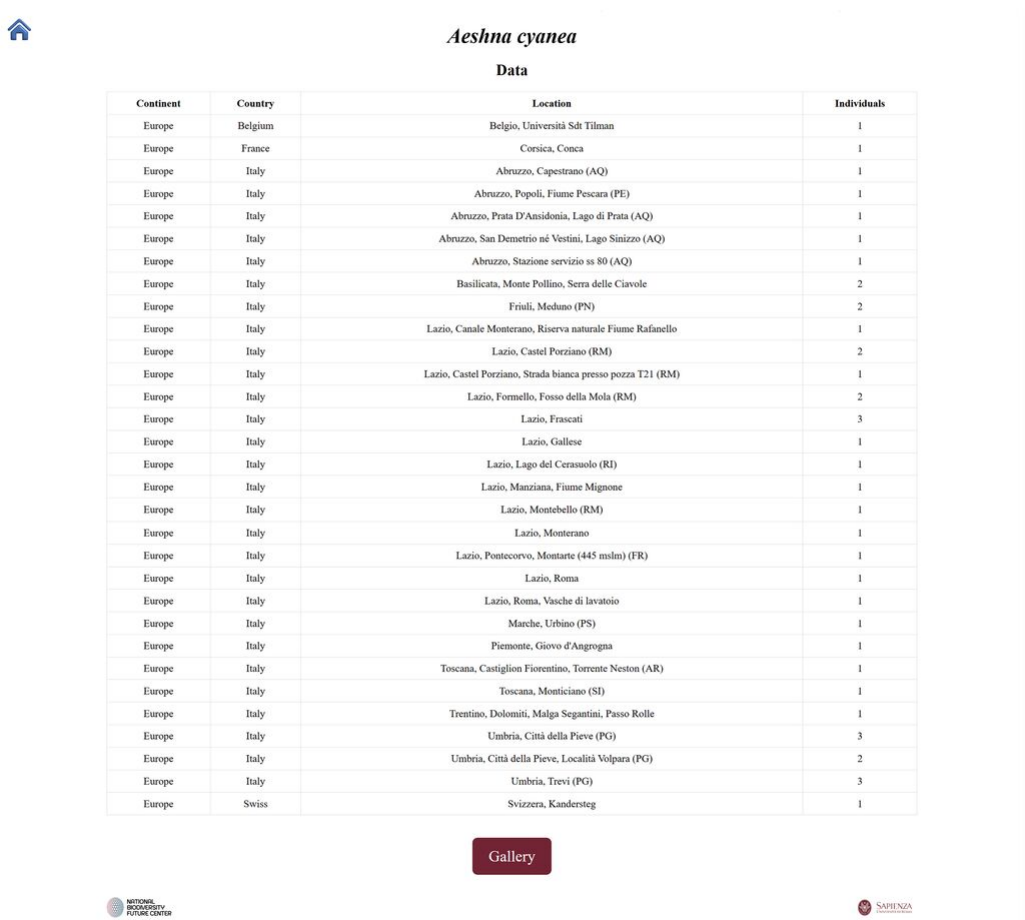
A dedicated web page for each species showing relevant data.

**Figure 4.**
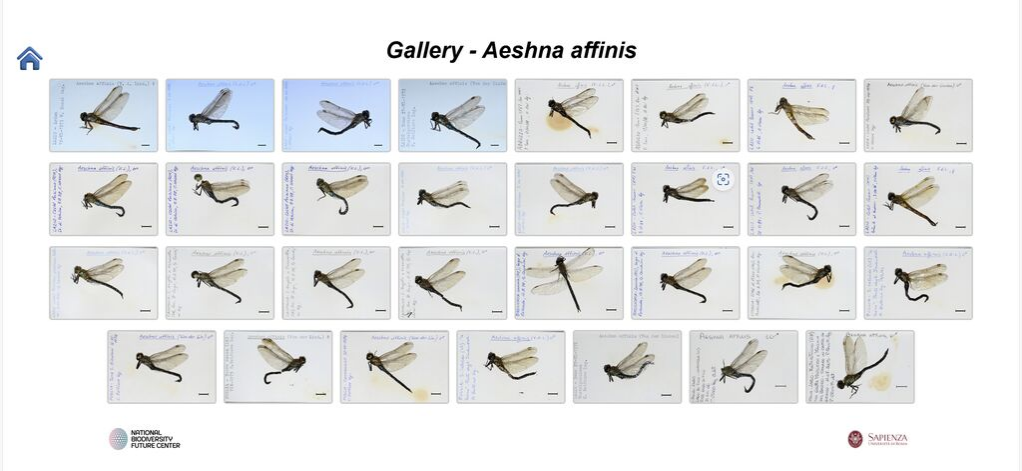
An example of a gallery with thumbnails of a single species.

**Figure 5.**
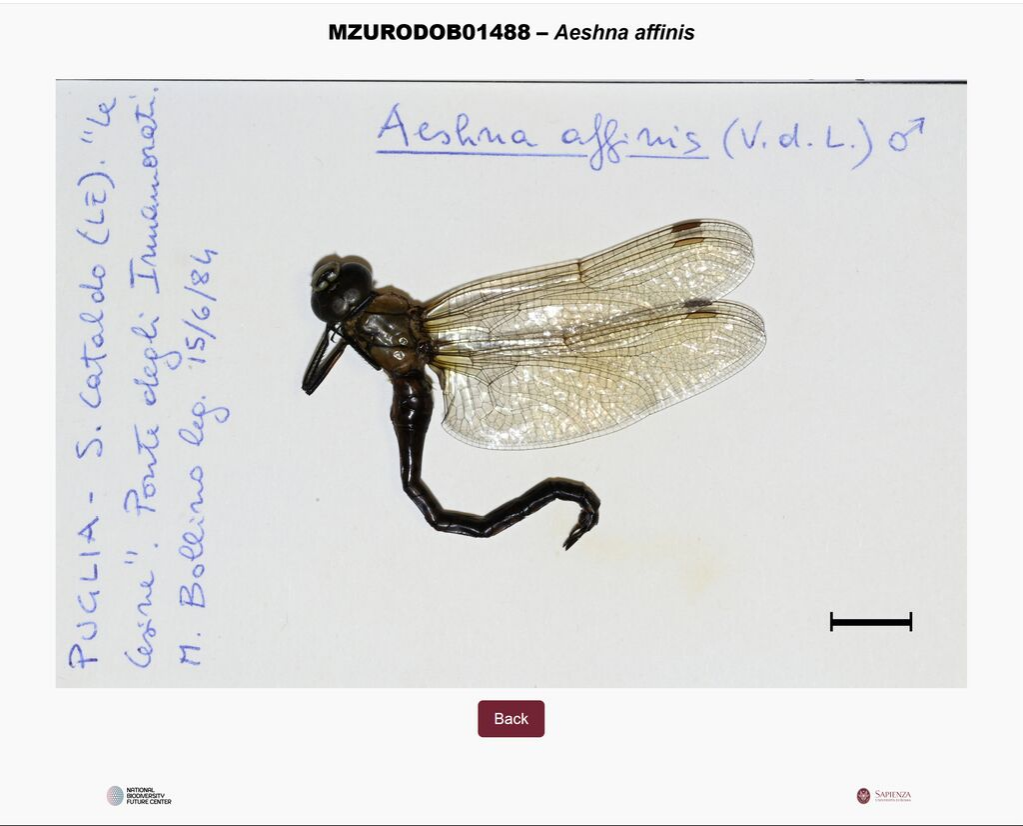
An example of the HTML page for a single specimen of a given species, showing the corresponding catalogue number.

**Second script**: Creates one HTML page per species, including a summary table of occurrences by locality (Fig. 3). Each page features a “Gallery” button that opens a thumbnail view of all individuals belonging to the species (Fig. 4). Clicking on a thumbnail leads to a detailed page with the enlarged specimen image, catalogue number and species name (Fig. 5).

**Figure 6.**
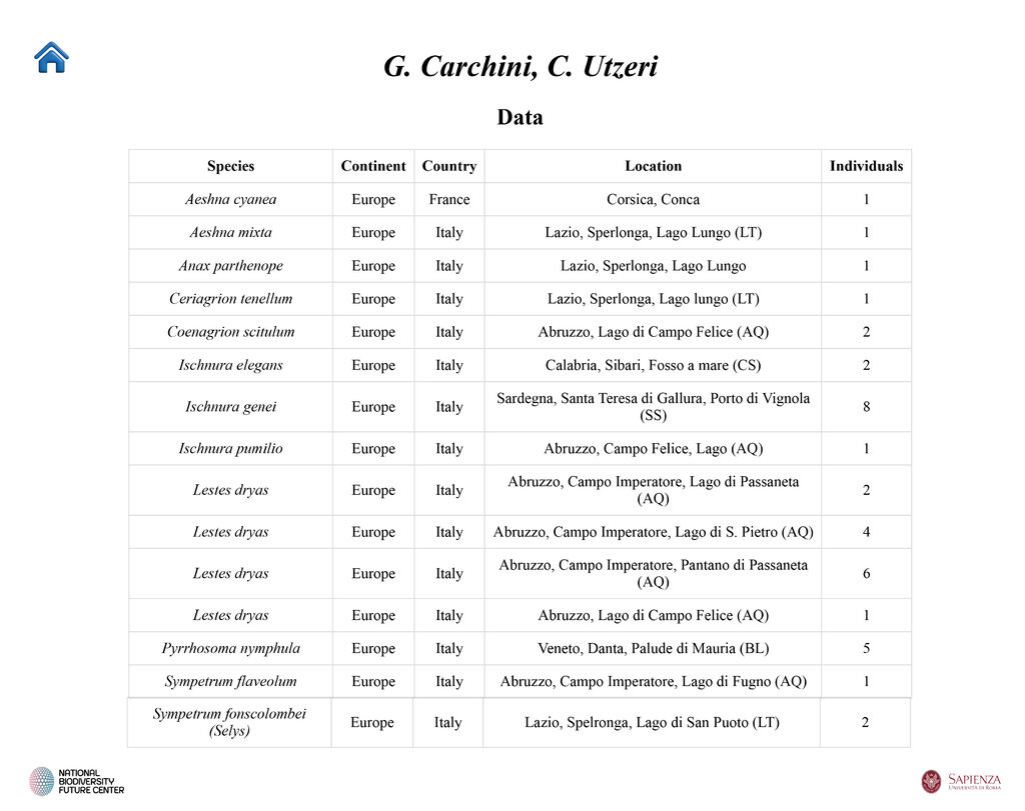
A web page devoted to each identifier, summarising the most relevant information.

**Third script**: ("Identifier"): Processes the Excel file to extract information on specimen identifiers and generates a table summarising data by species and associated collector(s) (Fig. 6).

**Figure 7.**
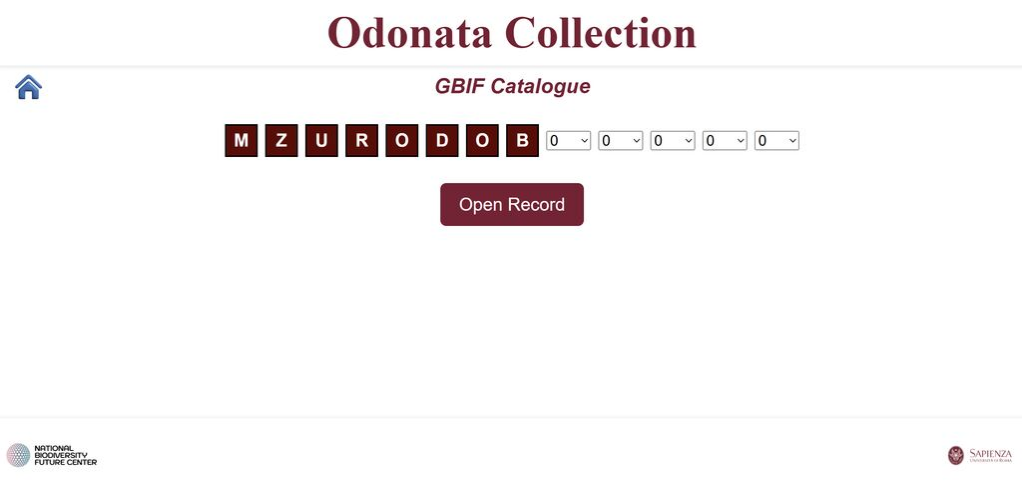
A web page for browsing the GBIF catalogue.

**Fourth script**: Generates an HTML interface that allows users to reconstruct GBIF-compliant codes for each sample using dropdown menus. The “Open Record” button links to the corresponding individual HTML file (Fig. 7).

**Figure 8.**
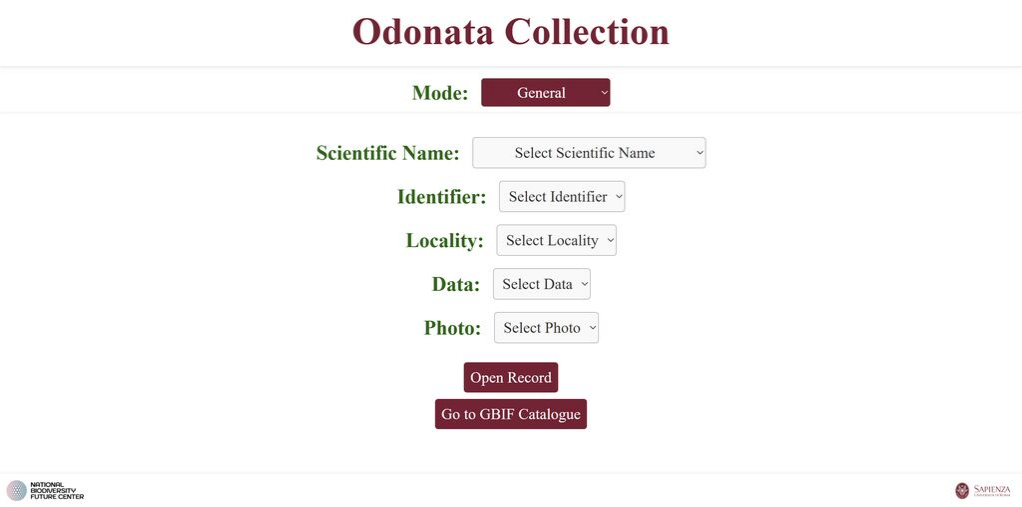
A web page featuring dropdown menus for exploring the full Odonata collection online.

**Fifth script**: Builds the main landing page (Home.html), providing three search options:

**1. General** search: via five linked dropdown menus (species, identifier, locality, date, photo) (Fig. 8). The “Open Record” button opens the corresponding individual HTML file (created with script 1).

**Figure 9.**
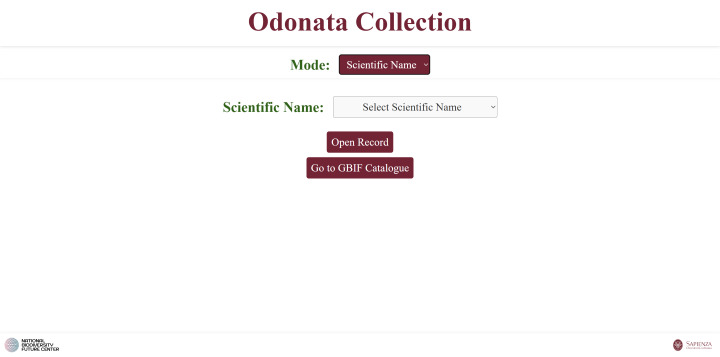
A web page that allows users to browse the catalogue by choosing individual species.

**2. Scientific name** search: via a single dropdown listing all species (Fig. 9). Each selection opens the HTML page generated for that species (created by Script 2).

**Figure 10.**
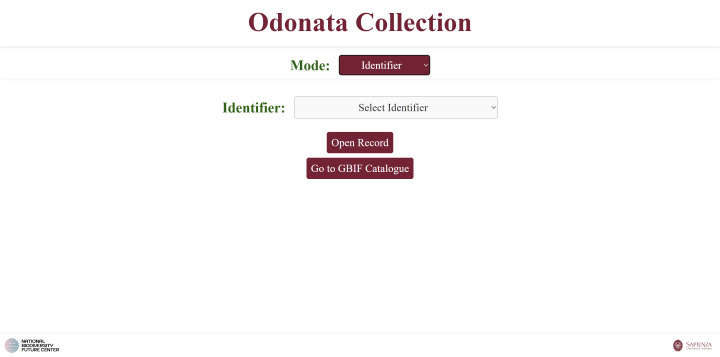
A web page that allows users to browse the catalogue, based on the identifier(s) of the specimens.

**3. Identifier** search: via a dropdown listing all identifiers or sets of identifiers (Fig. 10). Selecting one opens the specific HTML page dedicated to that identifier (created by Script 3).

In all cases, after selecting the desired criteria and clicking “Open Record,” users are directed to the corresponding HTML page with specimen data.

### Funding

This project was developed within the framework of the National Biodiversity Future Centre (NBFC), funded by the Italian Ministry of University and Research (PNRR, Missione 4 Componente 2, “Dalla ricerca all’impresa”, Investimento 1.4, project CN00000033).

## Sampling methods

### Study extent

**Figure 11.**
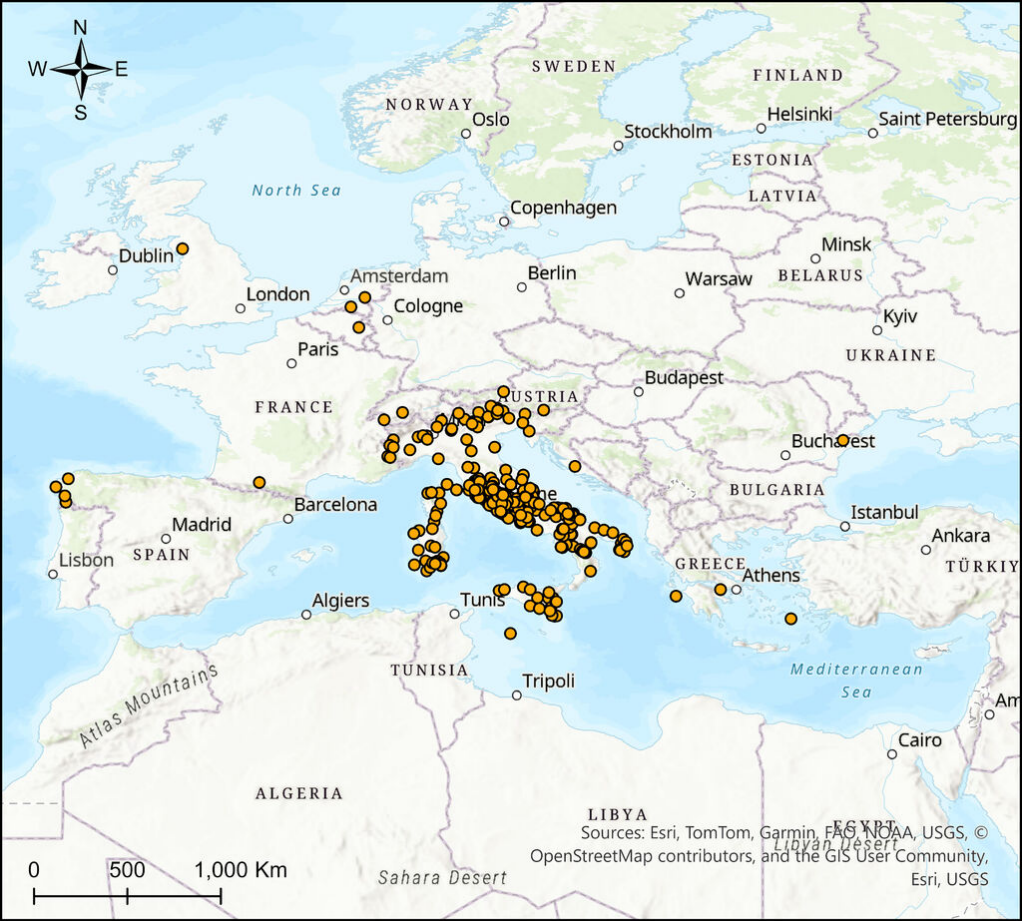
Dragonfly distribution map showing the specimens housed in the Odonata collection in the Museum of Zoology of Sapienza University of Rome.

The Odonata specimens in this collection were collected between 1936 and 2009 across 19 Italian regions and a few additional European locations. The most represented regions include Lazio (39% of samples), Apulia (14%) and Tuscany (9%). The collection sites encompass a wide range of freshwater habitats, from coastal wetlands to mountain streams; the 75 species represent more than 90% of the known Italian Odonata fauna. Most of the specimens were collected during targeted field campaigns in central and southern Italy (Fig. 11).

### Sampling description

The specimens were collected over several decades (1936–2009), mainly by hand netting and entomological trapping in various freshwater habitats across Italy and parts of Europe. Most specimens were collected by expert odonatologists during targeted fieldwork campaigns. Each specimen was mounted on cards and labelled with collection data, including locality, date, collector name and sex.

### Quality control

Specimens were reviewed and identified by qualified taxonomists. The digitisation included high-resolution photography and careful transcription of label data. A unique catalogue number was assigned to each individual or pair of specimens and metadata were verified before being uploaded to GBIF.

### Step description

- Specimens were mounted and labelled following standard entomological protocols;
- Labels were photographed with a Nikon Z7 camera under standardised lighting and framing;
- Each image was cropped and calibrated using automated scripts in Photoshop;
- Five custom Python scripts were developed to associate images with metadata, generate HTML interfaces and enable online consultation;
- Data were formatted according to Darwin Core standards and uploaded to GBIF;
- The digital archive is available online.

## Geographic coverage

### Description

The collection covers a broad geographic range, with specimens collected across 19 administrative regions of Italy (Table 1), as well as a few locations in other European countries, such as Switzerland. The most intensively sampled area is central Italy (57% of specimens). The habitats represented include a variety of freshwater ecosystems, from lowland wetlands and ponds to high-altitude streams, offering a comprehensive overview of the Italian Odonata biodiversity.

### Coordinates

36.6 and 46.7 Latitude; 6.6 and 18.5 Longitude.

## Taxonomic coverage

### Description

The collection includes 2406 Odonata specimens, representing 73 species and three genera classified into 11 families and 33 genera (Table 2). These belong to the two major suborders of Odonata (Table 3, Table 4):

- Zygoptera (damselflies): 28 species and one genus, 1,339 specimens (Table 3);
- Anisoptera (true dragonflies): 46 species and two genera, 1,067 specimens (Table 4).

The recorded taxa represent more than 90% of the known Italian odonatofauna (Carchini et al. 1985; La Porta et al. 2023). The most represented families are Libellulidae, Coenagrionidae and Aeshnidae.

### Taxa included

**Table:** 

Rank	Scientific Name	
kingdom	Animalia	
subkingdom	Eumetazoa	
phylum	Arthropoda	
class	Insecta	
order	Odonata	
suborder	Zygoptera	
family	Calopterygidae	
family	Coenagrionidae	
family	Lestidae	
family	Platycnemididae	
suborder	Anisoptera	
family	Aeshnidae	
family	Cordulegastridae	
family	Corduliidae	
family	Gomphidae	
family	Libellulidae	
family	Macromiidae	
family	Synthemistidae	

## Traits coverage

The collected data include a variety of morphological and ecological traits associated with each specimen. These traits have been recorded from the label data and include:

Body size: To ensure accurate measurement of body size, a 1 cm-long scale-bar was photographed alongside each specimen. This scale-bar was placed on the same cardboard as the specimen label and was used to calibrate the images for precise size measurement. The scale-bar images were then resampled to ensure uniform size reference across all photographs.

Sex: male/female, as indicated on the labels.

Ecological traits specimens were collected from different types of freshwater habitats, including wetlands, ponds, streams and rivers and across a remarkable altitudinal range (from lowland to mountain habitats).

Obviously, the dataset include detailed locality data, providing insights into species distribution across different regions in Italy and Europe.

Collection dates provide data on phenology allowing us to summarise seasonal patterns of occurrence, as species were collected throughout the year.

## Temporal coverage

### Notes

**Figure 12.**
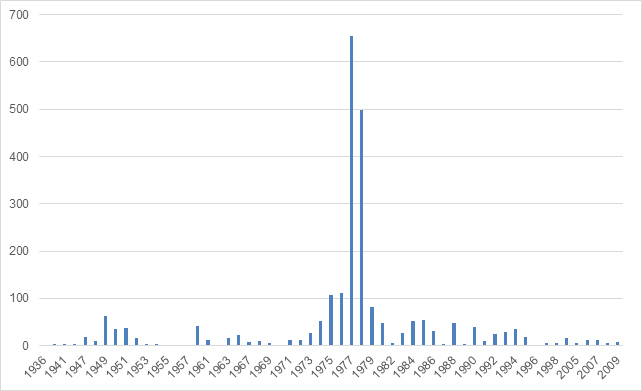
Specimens collected per year.

The specimens in this collection were collected over a span of several decades, from 1936 to 2009, providing valuable insights into the long-term biodiversity and distribution of Odonata in Italy. The collection reflects temporal patterns of species occurrence, with an increase in specimen numbers observed particularly in the late 1970s and a steady continuation of specimen collection through the 1980s and 2000s. This temporal coverage offers a unique perspective for studying trends in species distribution, population dynamics and the potential impacts of environmental changes over time (Fig. 12).

## Collection data

### Collection name

Odonata Collection of the Zoological Museum, Sapienza University of Rome (MZUR). This collection includes dragonfly specimens from across Italy and parts of Europe, collected over several decades. It is housed in the Zoological Museum of Sapienza University of Rome.

### Collection identifier

MZURODOB. Each specimen in the collection is assigned a unique identifier, which consists of a fixed part indicating the Zoological Museum of Sapienza University (MZUR) and the acronym of the Odonata collection (ODOB) (= MZURODOB) and a variable part (a five-digit number) that ensures the precise identification of each specimen.

### Parent collection identifier

MZUR. The Odonata collection is part of the broader entomological holdings of the Zoological Museum of Sapienza University.

### Specimen preservation method

The specimens are mounted on entomological cards and stored in protective entomological envelopes to prevent degradation and damage. These preservation methods ensure the integrity of the specimens over time, safeguarding their morphological and taxonomic characteristics. A standard procedure was followed to ensure optimal preservation of each specimen.

### Curatorial unit

The Odonata collection is managed and curated by the Zoological Museum of Sapienza University of Rome, under the supervision of entomologists and curators specialising in dragonfly species. The collection is regularly maintained and specimens are digitally catalogued, with detailed metadata for easy access and research purposes.

## Usage licence

### Usage licence

Creative Commons Public Domain Waiver (CC-Zero)

## Data resources

### Data package title

Odonata Collection of Zoology Museum - Sapienza University

### Resource link

https://doi.org/10.15468/hhq5g5

### Number of data sets

1

### Data set 1.

#### Data set name

Odonata Collection of the Zoological Museum of Sapienza University of Rome

#### Data format

Darwin Core occurrences

#### Character set

UTF-8

#### Download URL

https://cloud.gbif.org/eca/resource?r=odonati_mzur&v=1.11

#### Description

These data include occurrence records of Odonata (dragonflies and damselflies), curated at the Zoology Museum of Sapienza University (Chimenti 2025). The data include taxonomic identifications, geographic coordinates, collection dates and associated metadata following the Darwin Core standard.

**Data set 1.** 

Column label	Column description
occurrenceID	A unique identifier for the occurrence.
basisOfRecord	The specific nature of data record.
eventDate	Date in which the specimen was collected.
startDayOfYear	Day of the year.
endDayOfYear	Last day of event.
year	Year.
month	Month.
day	Day.
verbatimEventDate	Date in which the specimen was collected.
kingdom	Kingdom in which the taxon was classified.
phylum	Phylum in which the taxon was classified.
class	Class in which the taxon was classified.
order	Order in which the taxon was classified.
family	Family in which the taxon was classified.
genus	Genus in which the taxon was classified.
specificEpithet	The species part of a scientific name.
taxonRank	Taxonomic rank of the most specific name.
scientificName	The complete Latin name of the taxon, including genus, species and authorship, as assigned during identification.
higherClassification	Full taxonomic hierarchy above the species level.
identifiedBy	Who identified the organism.
nomenclaturalCode	The naming code used for the scientific name.
decimalLatitude	Latitude of the occurrence in decimal degrees.
decimalLongitude	Longitude of the occurrence in decimal degrees.
geodeticDatum	Geodetic datum of the geographic coordinates.
coordinateUncertaintyInMetres	Uncertainty (in metres) associated with the geographic coordinates.
verbatimCoordinates	Coordinates as originally recorded, in any format.
verbatimCoordinateSystem	The system used to express the original coordinates.
georeferencedDate	The date when the geographic coordinates were determined for the location.
georeferenceProtocol	How the georeferencing was done.
georeferenceSources	Sources consulted to assign the coordinates.
georeferenceVerificationStatus	The verification status of the georeference.
higherGeography	The broader geographic area that includes the location.
continent	Continent where the specimen was collected.
island	Island where the specimen was collected.
country	Country where the specimen was collected.
countryCode	Standardised code of the country.
stateProvince	Administrative region where the specimen was collected.
locality	Locality where the specimen was collected.
type	The nature of the resource ("PhysicalObject" for all records).
language	The language of the resource.
institutionID	The unique identifier for the institution.
collectionCode	Code of the collection.
catalogNumber	Number of the catalogue.
sex	The sex of the sample.
organismID	The unique ID for the organism or specimen.
individualCount	The number of individual presented present at the time of occurrence.
organismQuantity	A number or enumeration value for the quantity of organisms.
organismQuantityType	The type of quantification system used for the quantity of organisms.
samplingProtocol	Method used to collect the specimens.
preparations	Type of preparation.
otherCatalogNumbers	Other catalogue numbers.
associatedMedia	It contains links to photos of the specimens documented in the dataset.
lifeStage	Developmental stage of the organism.

## Figures and Tables

**Table 1.** Total number of species per administrative regions (Italian regions only).

**Regions**	**Number of specimens**
Lazio	933
Apulia	343
Tuscany	216
Abruzzo	196
Basilicata	113
Sardinia	103
Umbria	101
Sicily	57
Molise	52
Campania	48
Calabria	41
Lombardy	28
Trentino Alto Adige	22
Marche	21
Piedmont	19
Friuli Venezia Giulia	13
Veneto	12
Emilia Romagna	6
Liguria	1

**Table 2.** Number of specimens per genera, ranked by the highest numbers.

**Genus**	**Number of specimens**
Ischnura	450
Sympetrum	302
Coenagrion	277
Platycnemis	251
Aeshna	150
Lestes	150
Anax	128
Onychogomphus	122
Ceriagrion	95
Orthetrum	87
Calopteryx	74
Crocothemis	60
Trithemis	60
Cordulegaster	33
Somatochlora	26
Anaciaeschna	22
Pyrrhosoma	21
Sympecma	21
Oxygastra	15
Boyeria	12
Gomphus	11
Calcholestes	10
Cordulia	6
Leucorrhinia	4
Libellula	4
Cercion	3
Enallagma	3
Brachytron	2
Paragompus	2
Selysiothemis	2
Erythromma	1
Macromia	1

**Table 3.** Number of specimens for the of Zygoptera.

** Zygoptera **	
**Species or genera**	**Number of specimens**
*Chalcolestes parvidens* (Artobolevskii, 1929)	1
*Chalcolestes viridis* (Vander Linden, 1825)	9
*Calopteryx haemorrhoidalis* (Vander Linden, 1825)	3
*Calopteryx splendens* (Harris, 1780)	1
*Calopteryx virgo* (Linnaeus, 1758)	70
*Cercion lindenii* (Sélys-Longchamps, 1840)	3
*Ceriagrion tenellum* (Villers, 1789)	95
*Coenagrion* (Kirby, 1890)	1
*Coenagrion caerulescens* (Fonscolombe, 1838)	46
*Coenagrion castellani* (Roberts, 1948)	6
*Coenagrion hastulatum* (Charpentier, 1825)	3
*Coenagrion mercuriale* (Charpentier, 1840)	20
*Coenagrion puella* (Linnaeus, 1758)	123
*Coenagrion pulchellum* (Vander Linden, 1825)	51
*Coenagrion scitulum* (Rambur, 1842)	27
*Enallagma cyathigerum* (Charpentier, 1840)	3
*Erythromma viridulum* (Charpentier, 1840)	1
*Ischnura elegans* (Vander Linden, 1820)	381
*Ischnura genei* (Rambur, 1842)	55
*Ischnura pumilio* (Charpentier, 1825)	14
*Lestes barbarus* (Fabricius, 1798)	55
*Lestes dryas* (Kirby, 1890)	27
*Lestes sponsa* (Hansemann, 1823)	2
*Lestes virens* (Charpentier, 1825)	46
*Lestes viridis* (Vander Linden, 1825)	20
*Platycnemis pennipes* (Pallas, 1771)	251
*Pyrrhosoma nymphula* (Sulzer, 1776)	21
*Sympecma fusca* (Vander Linden, 1820)	21

**Table 4.** Number of specimens for the species of Anisoptera.

** Anisoptera **	
**Species or genera**	**Number of specimens**
*Aeshna affinis* (Vander Linden, 1820)	31
*Aeshna cyanea* (Muller, 1764)	43
*Aeshna juncea* (Linnaeus, 1758)	20
*Aeshna mixta* (Latreille, 1805)	55
*Anaciaeschna isosceles* (Muller, 1767)	22
*Anax* (Leach, 1815)	1
*Anax ephippiger* (Burmeister, 1839)	6
*Anax imperator* (Leach, 1815)	69
*Anax parthenope* (Sélys-Longchamps, 1839)	52
*Boyeria irene* (Fonscolombe, 1838)	12
*Brachytron hafniense* (Muller, 1764)	2
*Cordulegaster* (Leach, 1815)	3
*Cordulegaster annulatus* (Latreille, 1805)	2
*Cordulegaster bidentatus* (Sélys-Longchamps, 1843)	13
*Cordulegaster boltonii* (Donovan, 1807)	15
*Cordulia aenea* (Linnaeus, 1758)	7
*Crocothemis erythraea* (Brullé, 1832)	60
*Gomphus flavipes* (Charpentier, 1825)	1
*Gomphus vulgatissimus* (Linnaeus, 1758)	10
*Leucorrhinia caudalis* (Charpentier, 1840)	1
*Leucorrhinia dubia* (Vander Linden, 1825)	1
*Leucorrhinia pectoralis* (Charpentier, 1825)	2
*Libellula fulva* (Muller, 1764)	2
*Libellula quadrimaculata* (Linnaeus, 1758)	2
*Macromia splendens* (Pictet, 1843)	1
*Onychogomphus forcipatus* (Linnaeus, 1758)	96
*Onychogomphus uncatus* (Charpentier, 1840)	26
*Orthetrum albistylum* (Sélys-Longchamps, 1848)	2
*Orthetrum anceps* (Fabricius, 1798)	1
*Orthetrum brunneum* (Fonscolombe, 1837)	78
*Orthetrum cancellatum* (Linnaeus, 1758)	1
*Orthetrum coerulescens* (Fabricius, 1798)	3
*Orthetrum trinacria* (Sélys-Longchamps, 1841)	2
*Oxygastra curtisii* (Dale, 1834)	15
*Paragomphus genei* (Sélys-Longchamps, 1841)	2
*Selysiothemis nigra* (Vander Linden, 1825)	2
*Somatochlora flavomaculata* (Vander Linden, 1825)	7
*Somatochlora meridionalis* (Nielsen, 1935)	11
*Somatochlora metallica* (Vander Linden, 1825)	7
*Sympetrum danae* (Sulzer, 1776)	10
*Sympetrum depressiusculum* (Sélys, 1841)	3
*Sympetrum flaveolum* (Linnaeus, 1758)	28
*Sympetrum fonscolombii* (Sélys-Longchamps, 1840)	139
*Sympetrum meridionale* (Sélys-Longchamps, 1841)	12
*Sympetrum pedemontanum* (Müller in Allioni, 1766)	2
*Sympetrum sanguineum* (Muller, 1764)	6
*Sympetrum striolatum* (Charpentier, 1840)	102
*Trithemis annulata* (Palisot de Beauvais, 1805)	60
